# Impact of lenvatinib-induced proteinuria and renal dysfunction in patients with thyroid cancer

**DOI:** 10.3389/fonc.2023.1154771

**Published:** 2023-03-14

**Authors:** Yuma Shibutani, Shinya Suzuki, Atsunobu Sagara, Tomohiro Enokida, Susumu Okano, Takao Fujisawa, Fumiaki Sato, Tetsuro Yumoto, Motohiko Sano, Toshikatsu Kawasaki, Makoto Tahara

**Affiliations:** ^1^ Department of Pharmacy, National Cancer Center Hospital East, Kashiwa, Chiba, Japan; ^2^ Hoshi University School of Pharmacy and Pharmaceutical Sciences, Shinagawa, Tokyo, Japan; ^3^ Department of Head and Neck Medical Oncology, National Cancer Center Hospital East, Kashiwa, Japan

**Keywords:** thyroid cancer, lenvatinib, proteinuria, renal dysfunction, VEGF inhibitors

## Abstract

**Background:**

Proteinuria is the most frequent adverse event of lenvatinib use. However, the association between lenvatinib-induced proteinuria and renal dysfunction remains unclear.

**Methods:**

We retrospectively reviewed medical records of patients with thyroid cancer without proteinuria treated with lenvatinib as a first-line systemic therapy at the initiation of treatment to assess the association between lenvatinib-induced proteinuria and renal function and the risk factors for the development of ≥3+ proteinuria on a dipstick test. Proteinuria was assessed by the dipstick test throughout the treatment in all cases.

**Results:**

Of the 76 patients, 39 developed ≤2+ proteinuria (low proteinuria group) and 37 developed ≥3+ proteinuria (high proteinuria group). There was no significant difference in estimated glomerular filtration rate (eGFR) between high and low proteinuria groups at each time point, but there was a trend toward a significant decrease in eGFR of -9.3 ml/min/1.73 m^2^ in all patients after 2 years of treatment. The percentage of change in eGFR (ΔeGFR) significantly decreased in the high proteinuria group compared to that in the low proteinuria group (ΔeGFR: -6.8% vs. -17.2%, p=0.04). However, there was no significant difference in development of severe renal dysfunction with eGFR <30 ml/min/1.73 m^2^ between the two groups. Moreover, no patients permanently discontinued treatment because of renal dysfunction in both groups. Furthermore, renal function after completion of lenvatinib was reversible.

**Conclusions:**

There was no association between the degree of lenvatinib-induced proteinuria and renal function. Therefore, treatment should be continued with attention to renal function, regardless of the degree of proteinuria.

## 1 Introduction

Differentiated thyroid cancer (DTC) is the most common histologic type of thyroid cancer and surgery is the first choice of treatment, with postoperative radioiodine (RAI) therapy if necessary (1–3).. Lenvatinib is a multi-kinase inhibitor that blocks a variety of receptors, including vascular endothelial growth factor receptor (VEGFR) 1-3 (4–6). In the SELECT study, a global Phase III trial in RAI-refractory DTC, lenvatinib significantly improved progression-free survival to 18.3 months, compared to 3.6 months with a placebo (7). As a result, lenvatinib is recommended for use in patients with RAI-refractory DTC (8).

Proteinuria associated with anti-cancer drug use is mainly caused by damage to the glomerulus of the kidney by targeting VEGF (9, 10); for instance, bevacizumab, an anti-VEGF drug causes the event in a dose-dependent manner in 41%–63% of patients (11, 12). Lenvatinib-induced proteinuria is one of the most frequent adverse events, represented by 31% for all grades and 10% for grade ≥3 in the SELECT trial, and notably, occurs more frequently in Japanese patients than in others (7, 13).. However, protocols and strategies for monitoring and managing proteinuria induced by lenvatinib remain unestablished. According to the National Cancer Institute Common Terminology Criteria for Adverse Events (CTCAE) v4.0, which were used to evaluate adverse effects in the SELECT study, a proteinuria ≤2+ or less is defined as grade 2 proteinuria, but if a proteinuria ≤3+ develops, a 24-hour urine sample should be performed when proteinuria ≥3+ develops; a urine sample with >3.5 g/gCre is defined as a grade ≥3 proteinuria. Because this 24-hour urine sample test relies on the patient collecting overnight urine samples, which is difficult to test during outpatient therapy, lenvatinib was often withdrawn when proteinuria ≥3+ developed (14). Thus, the details of renal dysfunction with continued lenvatinib after the onset of proteinuria ≥3+ are unclear. Since lenvatinib was continued regardless of the degree of proteinuria in the absence of a rapid decrease in either eGFR or edema, and to not decrease the intensity of treatment at our hospital, we investigated the effect of the degree of proteinuria on renal function in this study.

## 2 Materials and methods

### 2.1 Participants

Seventy-six patients with thyroid cancer without proteinuria treated with lenvatinib as a first-line systemic therapy at the initiation of treatment at the National Cancer Center Hospital East from October 2011 to June 2021 were retrospectively reviewed. The data cutoff date was 31st October 2021, which is the period covered by the study. The management of proteinuria, including interruption or dose reduction of lenvatinib, was performed by the attending physician.

### 2.2 Study design and methods

This was a single-center, retrospective study conducted using medical records. The status of the development of proteinuria, change in estimated glomerular filtration rate (eGFR), and effect of the degree of proteinuria on the continuation of lenvatinib treatment were evaluated. To estimate the effect of lenvatinib-induced proteinuria on renal function, we compared two groups: the low proteinuria group, in which the participant developed ≤2+ proteinuria on dipstick, and the high proteinuria group with ≥3+ proteinuria on dipstick. The eGFR formula used was specific to the Japanese population (15). In all cases, proteinuria was assessed by a dipstick test throughout the treatment (16). Hypertension was defined as patients who had been diagnosed with hypertension prior to starting lenvatinib and were taking antihypertensive treatment. Patients were included if they had been diagnosed with diabetes mellitus prior to starting lenvatinib. Performance status (PS) was assessed with the Eastern Cooperative Oncology Group scale. The time point at which the laboratory test was performed was defined as follows: “pre-treatment” for the most recent laboratory value before lenvatinib treatment, “end of treatment” for the latest value during the treatment period, and “post treatment” for the latest value of observation period after end of treatment. Thus, the percentage change in eGFR (ΔeGFR) was calculated using the pre-treatment and post treatment values. The ΔeGFR was calculated using the formula used in the report by Iwasaki et al. (17).

### 2.3 Statistical analysis

For comparisons between high and low proteinuria groups, the univariate chi-square, Fisher’s exact, Mann–Whitney U, and Wilcoxon signed-rank tests were used. In addition, logistic regression analysis was used to identify risk factors for proteinuria ≥3+. Four factors were selected for logistic regression using multivariate analysis *via* the backward selection method. Statistical analyses were performed using SPSS ver.28.0 (IBM, Armonk, NY, USA), and p < 0.05 was considered statistically significant.

### 2.4 Ethical considerations

This study was approved according to its compliance with the Ethical Guidelines for Life Sciences and Medical Research Involving Human Subjects and was subjected to the ethical review procedures of the National Cancer Center. Compliance with the relevant guidelines was also ensured while performing research involving the participants during the original studies (Research Project No. 2022-115).

## 3 Results

### 3.1 Development of proteinuria and patient characteristics

Of the 76 patients, 39 patients developed ≤2+ proteinuria on the dipstick test during lenvatinib treatment and were categorized as the low proteinuria group, and 37 patients developed ≥3+ proteinuria on the dipstick test and were categorized as the high proteinuria group. The patients in the high proteinuria group had a significantly higher median age than those in the low proteinuria group (p=0.004), of which a significantly higher percentage was aged ≥65 years (p=0.01) (**Table 1**). In addition, the high proteinuria group was more significantly associated with a history of hypertension than the low proteinuria group (p=0.04). No significant difference in other items, including eGFR for pre-treatment, history of diabetes, use of antihypertensive medication, and lenvatinib treatment duration, were observed between the two groups.

**Table 1:** Patient characteristics.

	Low proteinuria group N=39	High proteinuria group N=37	p value
Sex (male), %	17 (44)	12 (33)	0.31^a^
Age, median (range)	65 (36–79)	70 (36–84)	0.004^b^
Age (≥65 years), %	20 (51)	29 (78)	0.01^a^
Body weight (kg), median (range)	54.3 (37.8–98.7)	54.7 (34.7–98.7)	0.80^d^
Treatment period (day), median (range)	447 (48–3280)	554 (46–3678)	0.27^c^
PS (≤1), %	38 (97)	36 (97)	0.74^d^
eGFR (ml/min/1.73m²) at pre-treatment, median (range)	73.7 (46.9–126.3)	65.4 (42.2–96.3)	0.12^b^
eGFR (<60 ml/min/1.73m²) at pre-treatment, %	9 (23)	13 (35)	0.24^a^
Hypertension, %	12 (30)	20 (54)	0.04^a^
CCB*, %	33 (84)	35 (94)	0.14^d^
ACEI/ARB*, %	31 (79)	32 (86)	0.41^a^
Diabetes, %	6 (15)	9 (24)	0.32^a^

Analysis Method: ^a^Chi-square test, ^b^Mann-Whitney U test, ^c^Two-sample t test, ^d^Fisher’s exact test

eGFR, estimated glomerular filtration rate; PS, Performance Status of Eastern Cooperative Oncology Group; CCB, calcium channel blocker; ACEI/ARB, Angiotensin-converting enzyme inhibitor/angiotensin II receptor blocker.

*Number of people taking oral medications during the treatment period

### 3.2 Temporal changes in eGFR and ΔeGFR

**Figure 1:**
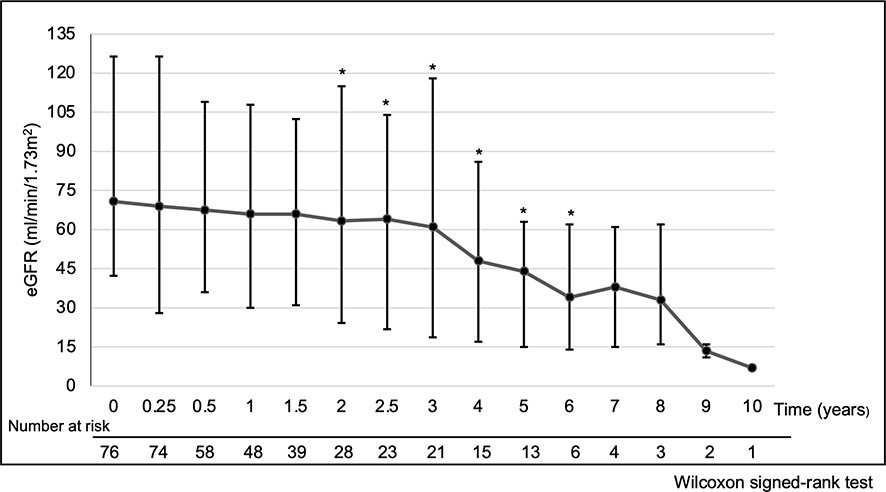
Temporal changes of eGFR. *p < 0.05, Time points showing a significant difference compared to pre-treatment eGFR. Analysis Method: Wilcoxon signed-rank test. Summary: eGFR at each time point compared to pre-treatment eGFR decreased significantly at 2 to 6 years of treatment. The median decreased eGFR change (and ΔeGFR) was -9.3 (-12.4), -16.7 (-27.9) and -30.5 ml/min/1.73 m^2^ (-32.2%) at 2, 4 and 6 years, respectively. Abbreviations: eGFR: estimated glomerular filtration rate, ΔeGFR: percentage of change in eGFR.

**Figure 2:**
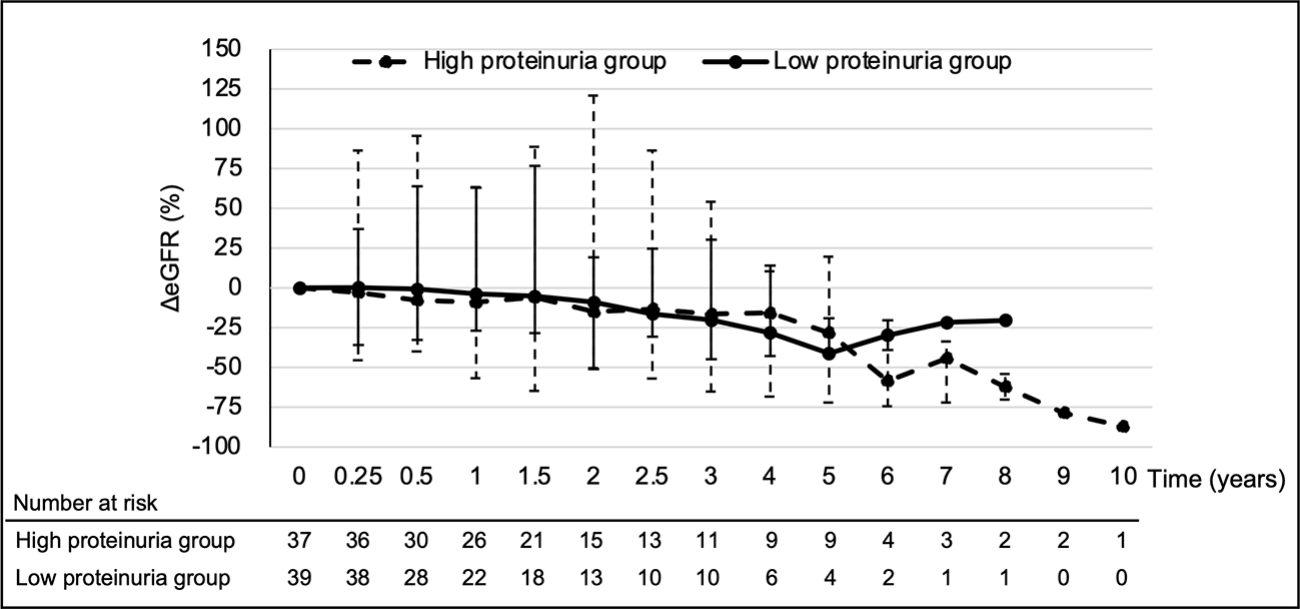
Temporal changes in ΔeGFR for each group. Analysis Method: Mann–Whitney U test. eGFR, estimated glomerular filtration rate; ΔeGFR, percentage of change in eGFR.

The median eGFR at each point in all 76 patients was calculated (**Figure 1**). eGFR at each time point decreased significantly at 2 to 6 years of treatment when compared to pre-treatment eGFR. The median decreased eGFR changes (and ΔeGFR) were -9.3 (-12.4), -16.7 (-27.9), and -30.5 ml/min/1.73 m^2^ (-32.2%) at 2, 4, and 6 years, respectively. However, the trends in ΔeGFR in the high and low proteinuria groups were not significantly different between the two groups at each point (**Figure 2**).

### 3.3 Changes in eGFR by time point in each group

**Figure 3:**
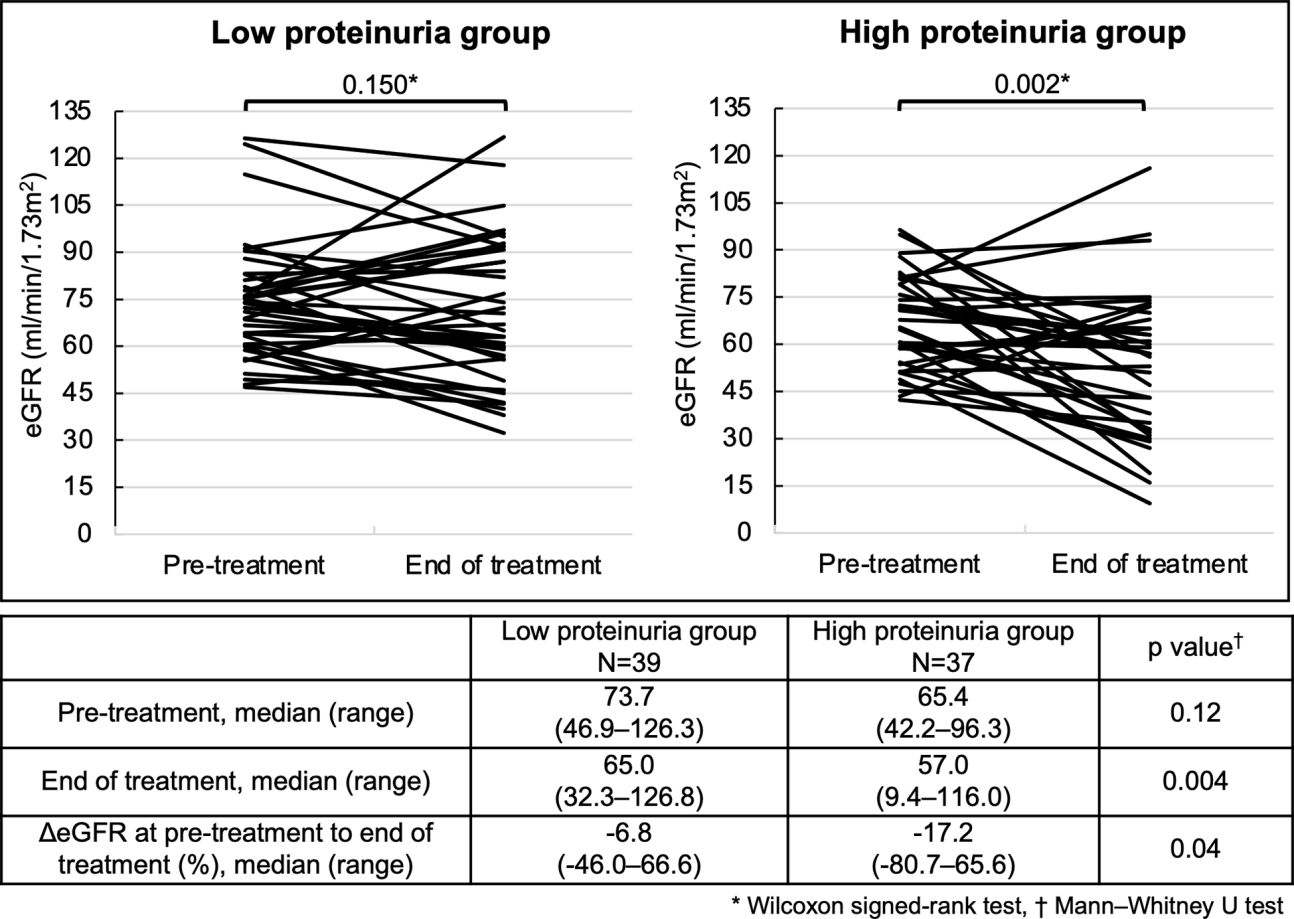
Changes in eGFR at pre-treatment to the end of treatment for each group. Analysis Method: *Wilcoxon signed-rank test, ^†^Mann–Whitney U test. eGFR, estimated glomerular filtration rate; ΔeGFR, percentage of change in eGFR.

There was no significant decrease in eGFR from pre-treatment to the end of treatment in the low proteinuria group (p=0.15) (**Figure 3**). However, the high proteinuria group showed a significant decrease in eGFR from pre-treatment to the end of treatment (p=0.002). There was no significant difference in eGFR at pre-treatment between the two groups (p=0.12). However, the high proteinuria group demonstrated a significant decrease of eGFR compared to the low proteinuria group (p=0.004). Furthermore, the high proteinuria group demonstrated a significant decrease in ΔeGFR from pre-treatment to the end of treatment compared with the low proteinuria group (-17.2% vs. -6.8%, p=0.04).

**Figure 4:**
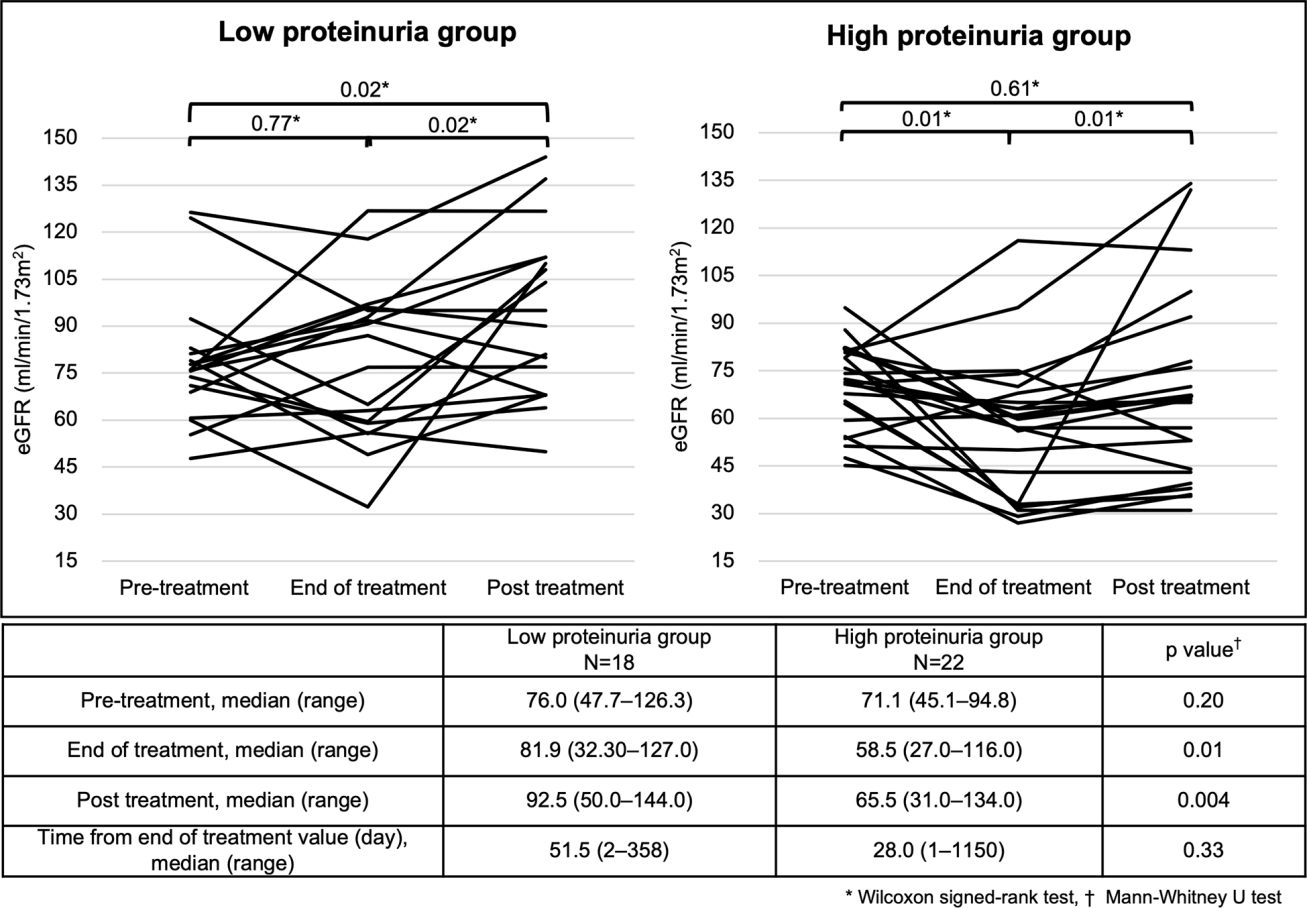
Changes in eGFR at different time points in each group. Analysis Method: *Wilcoxon signed-rank test, †; Mann–Whitney U test. eGFR, estimated glomerular filtration rate.

In both the low and high proteinuria groups, patients who were followed up after the end of treatment were identified, and renal function changes at pre-treatment, end of treatment, and post treatment are shown (**Figure 4**). There was no significant decrease in eGFR from pre-treatment to the end of treatment in the low proteinuria group (p=0.77). There was a significant increase in eGFR from the end of treatment to post treatment and from pre-treatment to post treatment in the low proteinuria group (p=0.02). In the high proteinuria group, there was a significant decrease in eGFR from pre-treatment to the end of treatment (p=0.01). However, eGFR was significantly increased from the end of treatment to post treatment (p=0.01). There was no significant difference in eGFR from pre-treatment to post treatment in the high proteinuria group (p=0.61). There was no significant difference in eGFR at pre-treatment between the low proteinuria and high proteinuria groups (p=0.20). However, eGFR was significantly lower for the high proteinuria group than for the low proteinuria group at the end of treatment and post treatment (end of treatment: p=0.01, post treatment: p=0.004).

### 3.4 Degree of proteinuria and its effect on renal function

**Figure 5:**
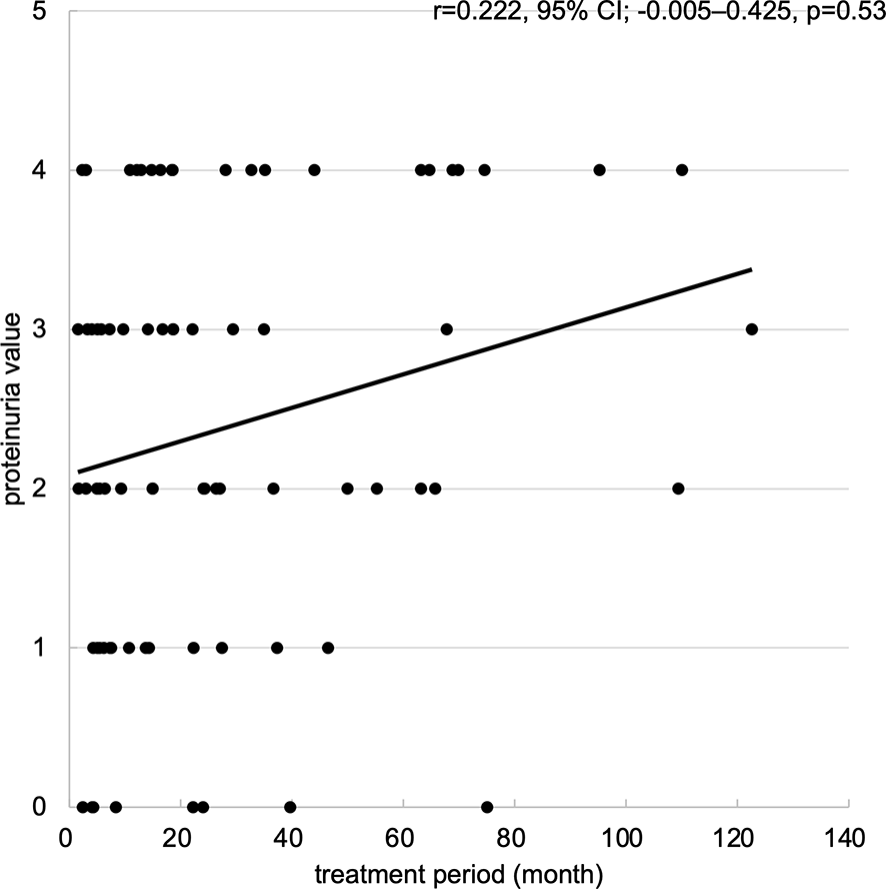
Scatter plots of maximum proteinuria values for treatment period. Scatter plots of maximum proteinuria values for treatment period. The horizontal axis represents the treatment period (month), and the vertical axis represents the maximum proteinuria value (from 0 to +4). Abbreviations: 95% CI: 95% confidence interval.

Regarding renal dysfunction corresponding to the degree of proteinuria, an eGFR measurement of 30-15 ml/min/1.73m^2^ was observed in 3 patients within the high proteinuria group, but there was no significant difference between the two groups (p=0.11) (**Table 2**). In addition, an eGFR measurement <15 ml/min/1.73 m^2^ occurred in 1 patient within the high proteinuria group, but there was no significant difference between the two groups (p=0.48). No patients required discontinuation of treatment or dialysis as a result of worsening renal function. Regarding the incidence of proteinuria, although the maximum proteinuria value during the treatment period tended to increase with longer treatment periods, there was no correlation (**Figure 5**).

**Table 2:** Degree of proteinuria and renal dysfunction.

	Low proteinuria group N=39	High proteinuria group N=37	p value
Onset date of maximal proteinuria, median (range)	67 (11–806)	130 (11–2839)	0.15^a^
eGFR 30-15 ml/min/1.73m^2^ at the end of treatment, %	0	3 (8)	0.11^b^
eGFR <15 ml/min/1.73m^2^ at the end of treatment, %	0	1 (2)	0.48^b^
Introduction of dialysis	0	0	^-^
Discontinuation of administration due to renal damage	0	0	^-^

Analysis Method: ^a^Mann–Whitney U test; ^b^Fisher’s exact test.

eGFR, estimated glomerular filtration rate.

### 3.5 Decrease in eGFR and risk factors

**Figure 6:**
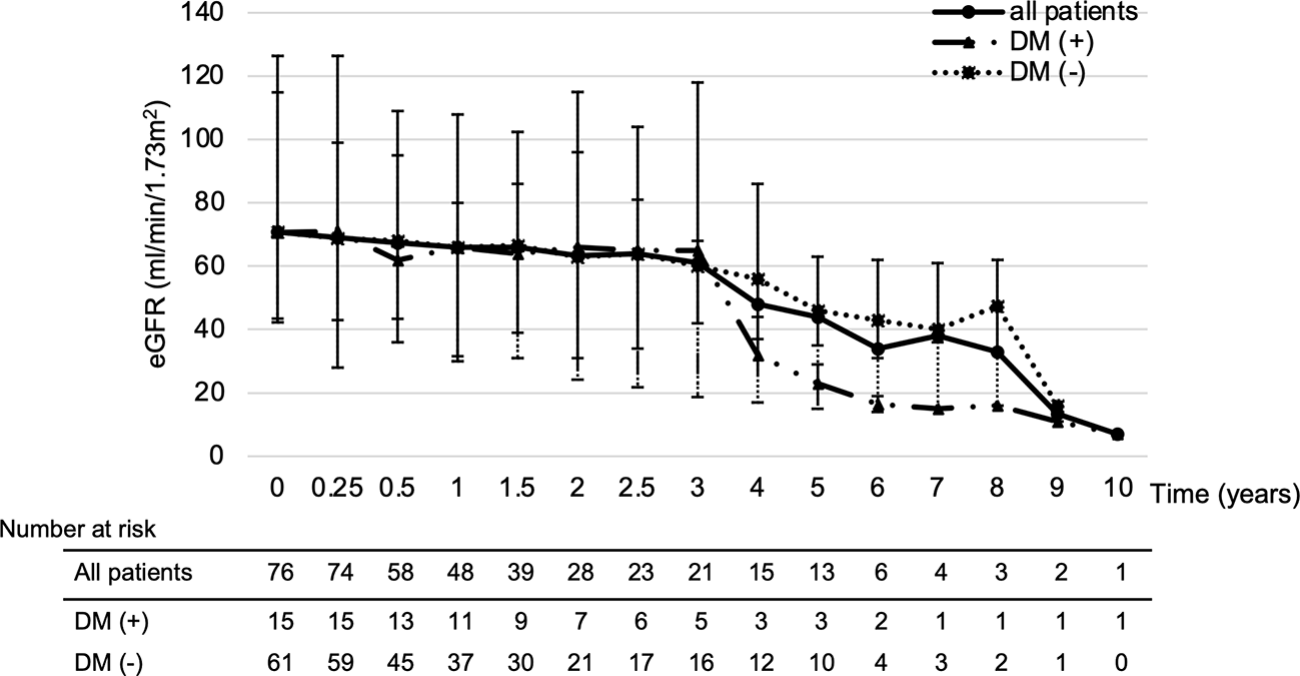
Impact of diabetes on eGFR changes. Looking at the effect of diabetes on eGFR, patients with a history of diabetes had a greatly reduced eGFR after 4 years of treatment. DM; diabetes mellitus, eGFR: estimated glomerular filtration rate.

The median ΔeGFR from baseline to the latest value of the treatment period for all patients was -7.6%. Therefore, a multivariate analysis of risk factors for patients with ΔeGFR >7.6% decrease revealed that diabetes (odds ratio [OR]; 5.28, 95% confidence interval [CI]; 1.27-21.85, p=0.02) and proteinuria ≥3+ (OR: 2.95, 95%CI; 1.06-8.17, p=0.03) were significantly related (**Table 3**). Thus, the change in eGFR in patients with a history of diabetes at the start of treatment showed that such patients had a lower eGFR after 4 years of lenvatinib treatment compared to both the overall population and the group without a history of diabetes (**Figure 6**).

**Table 3:** Decrease in eGFR and risk factors.

Factor	Univariate		Multivariate	
	Odds ratio (95% CI)	p value	Odds ratio (95% CI)	p value
Sex (male)	2.20 (0.85–5.69)	0.10	2.80 (0.98–8.03)	0.05
Age (≥65 years)	1.41 (0.55–3.63)	0.47	–	–
Diabetes	5.38 (1.37–21.04)	0.01	5.28 (1.27–21.85)	0.02
Hypertension	1.54 (0.61–3.85)	0.35	–	–
eGFR ≤60 ml/min/1.73m^2^ at pre-treatment	0.77 (0.28–2.09)	0.61	–	–
Proteinuria ≥3+	2.62 (1.04–6.63)	0.04	2.95 (1.06–8.17)	0.03
CCB	1.00 (0.23–4.32)	1.00	–	–
ACEI/ARB	1.76 (0.51–5.97)	0.36	–	–

Analysis Method: Logistic regression analysis

eGFR, estimated glomerular filtration rate; CCB, calcium channel blocker; ACEI/ARB, Angiotensin-converting enzyme inhibitor/angiotensin II receptor blocker.

The symbol “-” means that it is a step-wise excluded list item from the analysis.

## 4 Discussion

In this study, we examined the impact of the development of proteinuria ≥3+ on renal function during lenvatinib treatment. There was a significant decrease in eGFR in all patients from 2 years after the start of treatment, but it was clear that eGFR improved after the end of treatment. Furthermore, in the latest values of eGFR during treatment, the development of severe renal dysfunction or renal failure corresponding to a chronic kidney disease stage ≥G4 or higher was not significantly different between the two groups, and there was no permanent discontinuation of treatment due to renal dysfunction in both groups (18). Proteinuria induced by VEGF inhibitors, such as bevacizumab, increases in a dose-dependent manner, but is not associated with any renal dysfunction, such as decreased renal clearance and azotemia (19, 20). However, lenvatinib use causes proteinuria more frequently than use of other VEGF inhibitors because of the stronger VEGF inhibitory action of lenvatinib; furthermore, renal dysfunction occurred in 4.2% of patients in the SELECT study (7, 21). Patients with thyroid cancer treated with lenvatinib long-term have a significant decrease in eGFR after 2 years of treatment, which was also seen in this study (22). However, the change in ΔeGFR showed no significant difference between the high proteinuria and low proteinuria group at each time point, indicating that eGFR decreased regardless of the degree of proteinuria. In contrast, patients who ended treatment in both the high and low proteinuria groups showed a significant improvement in eGFR after the end of treatment, indicating that the decrease in eGFR during treatment was not due to irreversible renal dysfunction. In summary, although lenvatinib produces proteinuria more frequently than other VEGF inhibitors, eGFR declines after 2 years of treatment, regardless of the degree of proteinuria, and this decline in eGFR with lenvatinib is reversible. This suggests that lenvatinib-induced proteinuria may be only one of the phenotypes of renal dysfunction caused by VEGF inhibitors, and it is important to be aware of changes in renal function, regardless of the degree of proteinuria.

In general, risk factors for impaired renal function include age >65 years, diabetes mellitus, and hypertension (23). In addition, hypertension is a frequent side effect of lenvatinib use; and calcium channel blockers, angiotensin-converting enzyme inhibitors, and angiotensin II receptor blockers are often used to treat this side effect. However, these antihypertensive drugs can cause renal dysfunction (24–27). In this study, the multivariate analysis revealed that patients with a history of diabetes mellitus and patients who developed proteinuria ≥3+ were significantly associated with the risk of decrease in ΔeGFR. Unlike for other malignancies, the available treatment options for thyroid cancer are limited. Therefore, patients who respond to lenvatinib are more likely to use it long-term, and background factors such as medical history and age at the time of treatment initiation should also be considered. Diabetes mellitus can cause diabetic nephropathy, which manifests as a decrease in renal function over a long period (28), and increased proteinuria, which is strongly associated, along with decreased eGFR, with the risk of future end stage kidney disease in patients with diabetes mellitus (28, 29). In this study, renal failure was observed in one patient with a history of diabetes who developed proteinuria ≥3+. In addition, patients in the study with a history of diabetes showed a decrease in eGFR after 4 years of treatment compared to both the overall population and the population with no history of diabetes. These results suggest that eGFR and proteinuria should be carefully monitored in patients with a history of diabetes at the time of lenvatinib initiation. In addition, factors that were not identified in the multivariate analysis are reportedly associated with decreased renal function, suggesting that it is important to continue treatment with attention to the decrease in eGFR.

Two important limitations to this study must be considered. The first is that eGFR is a Japanese-specific value, which is affected by muscle mass. Therefore, eGFR may be overestimated in patients with reduced muscle mass, such as those with sarcopenia. Second, the protocol did not provide for temporary interruption or dose reduction of lenvatinib administration as a result of worsening proteinuria or renal function. Therefore, interruption or dose reduction was done at the discretion of the physician, and judgments based on the physician’s experience and knowledge may have affected renal function. However, there was no significant difference in ΔeGFR trends over time depending on the degree of proteinuria or in the development of severe renal dysfunction in the two groups. Thus, the results suggest that renal function should be monitored, and treatment continued cautiously regardless of the degree of urinary protein, but larger study are needed to confirm these findings.

## 5 Conclusion

Regardless of the degree of proteinuria, eGFR decreased after 2 years of lenvatinib treatment; however, the incidence of end stage renal dysfunction or renal failure was not significantly different between the two groups. In addition, patients with a history of diabetes mellitus should continue treatment with careful attention because it is a risk factor for a decrease in eGFR. Therefore, it is recommended to continue lenvatinib cautiously until more prospective data are published, paying attention to patient background and renal function.

## Data availability statement

The raw data supporting the conclusions of this article will be made available by the authors, without undue reservation.

## Ethics statement

The studies involving human participants were reviewed and approved by Shinya Suzuki, National Cancer Center Hospital East. Written informed consent for participation was not required for this study in accordance with the national legislation and the institutional requirements.

## Author contributions

YS, SS, and AS designed this concept, performed the statistical analyses, and wrote the manuscript. TE, SO, TF, FS, TY, MS, TK, and MT interpreted and discussed the data. All authors contributed to the article and approved the submitted version.
